# Supplementary material for: Transcriptome Analysis Reveals Vimentin-Induced Disruption of Cell–Cell Associations Augments Breast Cancer Cell Migration
Source: Cells. 2022 Dec 13;11(24):4035. doi: 10.3390/cells11244035 (PMC9776984; doi:10.3390/cells11244035)
Supplement: Supplementary file 1 [file cells-11-04035-s001.zip › DEGs and enrichment analysis.zip!/DEGs and enrichment analysis/WT_VIMvsVector KEGG/ALL/WT_VIMvsVector_ALL.pathway_map.zip!/WT_VIMvsVector_ALL.pathway_map/src/hsa00980.html]

hsa00980


- hsa:2947

- Down regulated genes

ENSG00000241720(-1.93726005347488)
- hsa:2946

- Down regulated genes

ENSG00000213366(-3.27565378318694)

- hsa:2947

- Down regulated genes

ENSG00000241720(-1.93726005347488)
- hsa:2946

- Down regulated genes

ENSG00000213366(-3.27565378318694)

- hsa:220

- Down regulated genes

ENSG00000259583(-2.65453851319078) ENSG00000184254(-1.95583461990274)

- hsa:2947

- Down regulated genes

ENSG00000241720(-1.93726005347488)
- hsa:2946

- Down regulated genes

ENSG00000213366(-3.27565378318694)

- hsa:2947

- Down regulated genes

ENSG00000241720(-1.93726005347488)
- hsa:2946

- Down regulated genes

ENSG00000213366(-3.27565378318694)

- hsa:2947

- Down regulated genes

ENSG00000241720(-1.93726005347488)
- hsa:2946

- Down regulated genes

ENSG00000213366(-3.27565378318694)

- hsa:2947

- Down regulated genes

ENSG00000241720(-1.93726005347488)
- hsa:2946

- Down regulated genes

ENSG00000213366(-3.27565378318694)

- hsa:2947

- Down regulated genes

ENSG00000241720(-1.93726005347488)
- hsa:2946

- Down regulated genes

ENSG00000213366(-3.27565378318694)

- hsa:2947

- Down regulated genes

ENSG00000241720(-1.93726005347488)
- hsa:2946

- Down regulated genes

ENSG00000213366(-3.27565378318694)

- hsa:2947

- Down regulated genes

ENSG00000241720(-1.93726005347488)
- hsa:2946

- Down regulated genes

ENSG00000213366(-3.27565378318694)

- hsa:2947

- Down regulated genes

ENSG00000241720(-1.93726005347488)
- hsa:2946

- Down regulated genes

ENSG00000213366(-3.27565378318694)

- hsa:2947

- Down regulated genes

ENSG00000241720(-1.93726005347488)
- hsa:2946

- Down regulated genes

ENSG00000213366(-3.27565378318694)

- hsa:2947

- Down regulated genes

ENSG00000241720(-1.93726005347488)
- hsa:2946

- Down regulated genes

ENSG00000213366(-3.27565378318694)

- hsa:2947

- Down regulated genes

ENSG00000241720(-1.93726005347488)
- hsa:2946

- Down regulated genes

ENSG00000213366(-3.27565378318694)

- hsa:2947

- Down regulated genes

ENSG00000241720(-1.93726005347488)
- hsa:2946

- Down regulated genes

ENSG00000213366(-3.27565378318694)

- hsa:1555

- Down regulated genes

ENSG00000256612(-3.39526895468382)

- hsa:2947

- Down regulated genes

ENSG00000241720(-1.93726005347488)
- hsa:2946

- Down regulated genes

ENSG00000213366(-3.27565378318694)

- hsa:2947

- Down regulated genes

ENSG00000241720(-1.93726005347488)
- hsa:2946

- Down regulated genes

ENSG00000213366(-3.27565378318694)

- hsa:1555

- Down regulated genes

ENSG00000256612(-3.39526895468382)

Close
